# Single and Multiple Doses of Seladelpar Decrease Diurnal Markers of Bile Acid Synthesis in Mice

**DOI:** 10.1155/ppar/5423221

**Published:** 2025-03-11

**Authors:** Edward E. Cable, Jeffrey W. Stebbins, Jeff D. Johnson, Yun-Jung Choi, Jiangao Song, Sole Gatto, Matthew Onorato, Charles A. McWherter

**Affiliations:** ^1^CymaBay Therapeutics Inc., Fremont, California, USA; ^2^Monoceros Biosystems LLC, San Diego, California, USA

**Keywords:** bile metabolism, circadian rhythm, PPAR, PPAR delta, seladelpar

## Abstract

Peroxisome proliferator–activated receptors (PPARs) modulate bile metabolism and are important therapeutic options in cholestatic diseases. This study was aimed at understanding the effects of single and multiple doses of seladelpar, a PPAR*δ* (peroxisome proliferator–activated receptor delta) agonist, on plasma C4 (a freely diffusible metabolite accepted as a proxy for de novo bile acid biosynthesis), Fibroblast Growth Factor 21 (Fgf21), and gene expression changes in the liver of male and female mice. C57BL/6 mice were treated with seladelpar 10 mg/kg/day or vehicle through oral gavage before lights out on Day 1 (single dose) or from Day 1 to Day 7 (multiple doses). Liver samples were obtained at 0, 1, 2, 4, 8, 12, 16, and 24 h postdosing, and plasma C4 and Fgf21 levels were measured. In vehicle-treated mice, C4 levels were higher in the dark cycle compared to the light cycle, with higher levels in females than in males. Plasma Fgf21 did not vary substantially over the dark–light cycle or show a sex-specific expression pattern. Seladelpar treatment significantly reduced plasma C4 and increased Fgf21 levels in both sexes, which coincided with a decrease in cholesterol 7*α*-hydroxylase mRNA and an increase in *Fgf21* mRNA in the livers. Untargeted RNA sequencing revealed a strong correlation between the genes differentially expressed after single- and multiple-dose seladelpar treatment. PPAR-responsive genes, including pyruvate dehydrogenase kinase 4, acyl-CoA thioesterase 2, and angiopoietin-like 4, were upregulated. No changes in nuclear receptors, clock genes, and sex-specific genes were observed. Overall, these results are consistent with a model where seladelpar treatment reduces bile acid synthesis by upregulating Fgf21 and modulating other PPAR-responsive genes.

## 1. Introduction

Bile acids play a key role in energy homeostasis, and their rate of production from cholesterol in the liver is regulated primarily by the expression of the rate-limiting enzyme cholesterol 7*α*-hydroxylase (Cyp7a1) [1, 2]. Bile acid metabolism in mice is closely linked to circadian rhythms [3–6] and relies upon environmental cues such as the light–dark cycle and fasting-feeding patterns [7, 8]. The molecular circadian network is driven by two core clock genes (circadian locomotor output cycles kaput (*Clock*) and basic helix-loop-helix ARNT-like 1 (*Bmal1*)) and their downstream targets, including the Period Circadian Regulator 1 and 2 (*Per1* and *Per2*) and the Cryptochrome Circadian Regulator 1 and 2 (*Cry1* and *Cry2*) [9]. In humans, bile acid metabolism also exhibits a diurnal rhythm. C4, a freely diffusible metabolite accepted as a proxy for de novo bile acid biosynthesis, peaks in the circulation after food intake during the day in humans and declines at night [3, 10]. In contrast, in animals, bile acid synthesis peaks at night and declines during the day due to their nocturnal feeding pattern. Consequently, the *Cyp7a1* gene also displays a strong diurnal expression pattern [11].

Disturbances in bile acid metabolism and disposition can cause liver diseases, such as primary biliary cholangitis [12], primary sclerosing cholangitis [13], and metabolic dysfunction-associated steatotic liver disease [14]. Despite their different etiologies, these diseases impair bile flow, leading to systemic bile accumulation and liver damage. Peroxisome proliferator–activated receptors (PPARs), a family of nuclear receptors, are an important therapeutic target for the treatment of liver diseases as they play a key role in bile metabolism [15, 16] and regulate the crosstalk between circadian rhythms and liver metabolic pathways [17]. Peroxisome proliferator–activated receptor delta (PPAR*δ*) has recently been identified as a novel therapeutic target for bile acid metabolism–related diseases. PPAR*δ* is expressed by all major cell types of the liver [18, 19], has a diurnal expression pattern, and also regulates bile acid homeostasis. A previous study demonstrated that knockout of the *Pparδ* gene in mice attenuates hepatic expression of lipogenic genes (acetyl-CoA carboxylase (*Acc1* and *Acc2*), fatty acid synthase (*Fasn*), and stearoyl-CoA 1 (*Scd1*)) during the dark cycle, demonstrating a crucial role of *Pparδ* in the diurnal regulation of lipogenic genes [20]. Additionally, daytime-restricted feeding reversed the expression pattern of *Pparδ* and lipogenic genes [20], demonstrating that *PPARδ* expression is regulated by food intake, not the cellular core clock mechanism.

Seladelpar (MBX-8025), a novel, potent, selective PPAR*δ* agonist, has shown promising results in clinical trials in patients with primary biliary cholangitis [21–23]. Preclinical studies with seladelpar have shown that it represses *Cyp7a1* expression and plasma C4 levels by inducing a negative regulator of bile synthesis, fibroblast growth factor 21 (Fgf21), establishing the crucial role of Fgf21 in PPAR*δ*-mediated *Cyp7a1* repression [24]. However, the diurnal rhythm of bile metabolism, bile metabolism genes, sex-specific effects, and the effects of a selective exogenous ligand remain to be elucidated. This study was conducted to understand the effects of single and multiple doses of seladelpar on plasma C4 and Fgf21 levels and global gene expression changes in the liver of male and female mice.

## 2. Materials and Methods

### 2.1. Animals and Treatment

The study was carried out at HD Biosciences Co. Ltd., Shanghai, China. Seven-week-old C57BL/6 mice were maintained for at least 5 days to acclimate before starting the study. The mice were maintained on a 12-h dark and 12-h light cycle (reverse cycle) and were given free access to a standard rodent diet and water ad libitum. The reversed light–dark cycle was implemented to more easily allow for the seladelpar dose to occur prior to eating at the beginning of the mice's awake/active cycle to mimic human morning dosing. The Animal Care and Use Committee of HD Biosciences reviewed and approved the study protocol.

**Figure 1:**
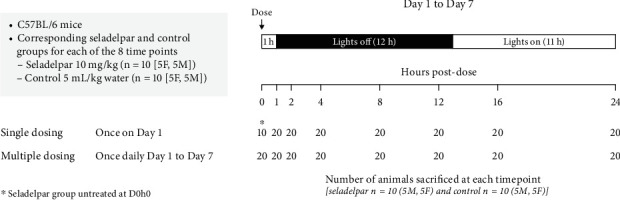
Study design. C57BL/6 mice were maintained on a reverse 12-h dark and 12-h lights-on cycle. Seladelpar 10 mg/kg/day or vehicle (5 mL/kg of water) was administered to mice before lights off (9 AM) via oral gavage once on Day 1 (single dose) or once daily from Day 1 to Day 7 (multiple doses). On Day 1 and Day 7, mice were sacrificed at 0, 1, 2, 4, 8, 12, 16, and 24 h postdose. Each seladelpar and control group had 10 mice (5 M and 5 F) for each time point on Day 1 and Day 7. D0h0, 0 h untreated; F, female; h, hours; M, male.

Mice were weighed and randomly assigned to control or seladelpar treatment groups (n =10 per group, with five males [weighing approximately 22–26 g] and five females [weighing approximately 17–20 g]), and body weights were monitored throughout the study. The sample size was based on preliminary data. Seladelpar 10 mg/kg (MBX-8025; CymaBay Therapeutics Inc.) was administered to mice once on Day 1 (single dose) or once daily from Day 1 to Day 7 (multiple doses). The corresponding group of control animals received 5 mL/kg of water (vehicle). Seladelpar or vehicle was administered to mice by oral gavage 1 h before lights out (9 AM). A schematic representation of the study design is provided in Figure 1.

On Day 1 and Day 7, mice were sacrificed (euthanized by CO_2_) at 0, 1, 2, 4, 8, 12, 16, and 24 h postdosing. Each time point for Day 1 and Day 7 consisted of 10 animals, with five males and five females. Blood was collected by cardiac puncture, transferred into K_2_-EDTA-coated tubes, and immediately kept on ice. Tubes containing blood were centrifuged at 4000 rpm for 10 min at 4°C to yield plasma. The liver, kidney, and spleen were harvested, transferred into RNase-free tubes, and snap-frozen in liquid nitrogen immediately. All samples were stored at −80°C until analysis.

### 2.2. Plasma C4 Measurements

C4 levels were measured in the plasma using tandem liquid chromatography-mass spectrometry at Quintara Discovery (Hayward, CA), as described previously [25], using an Exion UHPLC, with the column equilibrated at 40°C and a Sciex Qtrap 6500+ detector. Plasma samples were prepared by spiking with the respective internal standard before precipitation with acetonitrile. The precipitated samples were centrifuged, and an aliquot from the supernatant was used for the analysis. Standards were prepared using undiluted blank mouse plasma and were subjected to the same extraction methods. C4 was resolved using a Phenomenex Luna C18(2) 100 × 2 mm, 5 *μ*m column, and a mobile phase of water (A) and methanol (B), both with 0.1% acetic acid with the gradient (B% [*t* (min)]: 50 [0], 50 [0.2], 95 [0.5], 100 [5.5], and 50 [5.6]) using d_7_-C4 as an internal standard.

### 2.3. Plasma Fgf21 Measurements

Fgf21 levels were measured in plasma samples using a mouse/rat Fgf21 Quantikine ELISA kit (R&D Systems, Catalog No. MF2100), according to the manufacturer's instructions.

### 2.4. RNA Sequencing (RNA-seq) in Liver

Total RNA was extracted from 50 to 100 mg of frozen liver. RNA concentration and purity were measured (*n* = 5 males and 5 females for each time point), and 25 ng of RNA from each animal was used to create a pooled RNA sample that was subsequently used to create a single cDNA library. For selected samples, both pooled and individual animal samples were used to make libraries. Selected data are shown in the supporting information (Figure S1). Libraries were prepared from 100 ng of total RNA using a Truseq stranded mRNA library kit (Illumina, Catalog No. 20020595). One hundred nanograms of the pooled 125 ng sample produced a single data point using equal RNA amounts from five separate animals. Sequencing was performed on the Novaseq S4 platform according to the protocol provided by the manufacturer. Sequencing quality control was performed with FastQC v0.11.8. RNA-seq reads were trimmed, and low-quality reads were removed using Trimgalore v0.6.3_dev (https://www.bioinformatics.babraham.ac.uk/projects/trim_galore/) with the “paired” parameter and a length of 150 bps. The trimmed reads were aligned to the reference mouse genome GRCm38.94 (*Mus musculus* Ensembl mm10 sequence) using Spliced Transcripts Alignment to a Reference (STAR) v2.5.3a [26] with the produced BAM (Binary Alignment Map) files sorted by coordinate using the option “--outSAMtype BAM SortedByCoordinate.” Raw data were quantified at the gene level using STAR with options “--quantMode GeneCounts” and “--sjdbGTFfile” with gene models in Gene Transfer Format (GTF) obtained from the mouse Ensembl release 94. Alignment quality control and read mapping statistics were obtained from Picard tools v2.20.3 using the function “CollectMultipleMetrics” (http://broadinstitute.github.io/picard/). Gene level transcripts per million (TPM) reads quantification was done using RSEM (RNA-Seq by Expectation-Maximization) v1.3.1 [27]. All samples had more than 75% of the reads uniquely mapped to the mouse genome, and each sample had at least 25 million uniquely mapped reads.

### 2.5. Statistical Analysis

Statistical analyses were performed using JMP (v17). Individuals performing the liquid chromatography–mass spectrometry (LC–MS) or ELISA analyses were not aware of the study endpoints. Plasma C4 and Fgf21 data were analyzed by repeated measures over time. Based on previous data, seladelpar was expected to decrease C4 and increase Fgf21 [24]. If the primary endpoint data of this study replicated these known results in addition to the data correlating with known expression patterns of clock genes, the study was determined to function correctly. If between-subjects were tested significant (*F* < 0.05), then individual time points were analyzed by repeated measures multiple analysis of variance and Tukey–Kramer post hoc analysis. Data were expressed as mean ± standard error mean (SEM). Significant differences in the treatment or sex are described in the figure legends.

Differential gene expression analysis from the RNA-seq data was conducted using linear models for microarray data (Limma [3.40.6]) R package [28]. The samples were averaged for the individual genes analyzed from the RNA-seq data, and no additional statistical tests were performed other than the quality control and read mapping statistics described above. To visualize the similarities or differences in groups, all selected genes plotted individually had the same *y*-axis.

## 3. Results

### 3.1. C4, Not Fgf21, Followed a Diurnal and Sex-Specific Pattern

**Figure 2:**
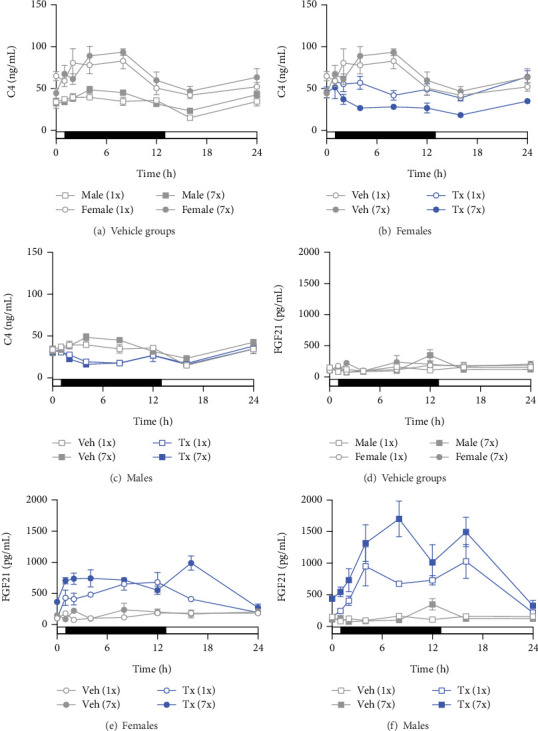
Plasma C4 and Fgf21 levels in control and seladelpar-treated mice. Plasma C4 and Fgf21 levels in (a, d) control (gray circles for female mice and gray squares for male mice), single, or multiple doses of seladelpar-treated (b, e) female (blue circles) and (c, f) male (blue squares) mice. Open symbols denote a single dose of seladelpar treatment or vehicle administration on Day 1 (1x). Multiple doses of seladelpar or vehicle administration from Day 1 to Day 7 (7x) are denoted using closed symbols. Data were expressed as mean ± SEM. h, hours; SEM, standard error of the mean; Tx, treated; Veh, vehicle.

Plasma C4 (a biomarker of de novo bile acid synthesis) and Fgf21 (a negative regulator of bile synthesis) levels were examined to understand the effects of seladelpar on bile homeostasis. Plasma C4 levels were higher in control female mice compared to male mice (Figure 2(a)). In both female and male mice, plasma C4 levels were higher in the dark cycle compared to the light cycle (Figure 2(a)). Plasma Fgf21 levels did not exhibit a sex-specific pattern or change over the dark–light cycle in control female or male mice (Figure 2(d)).

### 3.2. Single and Multiple Doses of Seladelpar Decreased Plasma C4 and Increased Plasma Fgf21 Levels

Single and multiple doses of seladelpar reduced the high plasma C4 levels observed in control mice of both sexes (Figures 2(b) and 2(c)). The reduction of C4 levels by seladelpar was significant at 2 h and remained so through 8 h postdosing at both Day 1 and Day 7. In male mice, single and multiple doses of seladelpar decreased plasma C4 levels in a similar manner (Figure 2(c)). In female mice, a single dose of seladelpar decreased C4 only at 8 h, while multiple doses of seladelpar significantly decreased plasma C4 levels from 2 to 24 h (Figure 2(b)). Seladelpar treatment also increased plasma Fgf21 levels in both female (Figure 2(e)) and male (Figure 2(f)) mice. In males, a significant increase in plasma Fgf21 was observed following multiple doses of seladelpar (Figure 2(f)).

### 3.3. Seladelpar-Induced Plasma C4 Reduction Coincided With Changes in Cyp7a1 and Fgf21 mRNA Expression in the Liver

**Figure 3:**
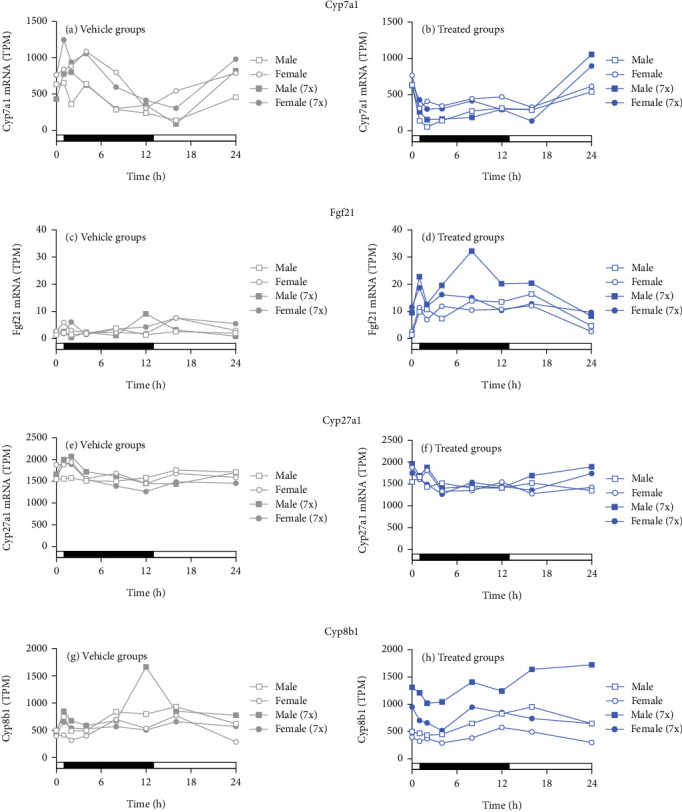
Seladelpar-induced changes in *Cyp7a1* and *Fgf21*. *Cyp7a1*, *Fgf21*, *Cyp27a1*, and *Cyp8b1* expression in (a, c, e, g) control (gray circles for female mice and gray squares for male mice) and (b, d, f, h) single- or multiple-dose seladelpar-treated female or male mice (blue circles for females and blue squares for males). Open symbols denote a single dose of seladelpar treatment or vehicle administration on Day 1 (1x). Multiple doses of seladelpar or vehicle administration from Day 1 to Day 7 (7x) are denoted using closed symbols. h, hours; TPM, transcripts per million.

To evaluate the correlation of plasma C4 and Fgf21 levels with *Cyp7a1* (which encodes the rate-limiting enzyme in the bile acid synthesis) and *Fgf21*, gene expression changes were assessed using the genome-wide RNA-seq data. In control mice, liver *Cyp7a1* expression coincided with plasma C4 levels, which followed a diurnal rhythm of increased expression during the dark cycle and decreased expression during the light cycle (Figure 3(a)). Notably, higher plasma C4 levels were observed in control female mice compared to male mice (Figure 2(a)), but greater repression of *Cyp7a1* mRNA expression was observed in male mice (Figure 3(a)). Single and multiple doses of seladelpar treatment similarly reduced *Cyp7a1* mRNA expression in both female and male mice (Figure 3(b)). Though multiple doses of seladelpar treatment demonstrated increased effects on plasma C4 in female mice (Figure 2(b)), repeat dosing did not differ meaningfully from single doses for *Cyp7a1* expression in either sex.

*Fgf21* liver mRNA levels in control female and male mice did not vary over dark–light cycles (Figure 3(c)), consistent with plasma Fgf21 levels (Figure 2(d)). Single and multiple doses of seladelpar treatment increased liver *Fgf21* (Figure 3(d)) expression in both sexes. Moreover, multiple-dose seladelpar treatment had an additive effect on plasma Fgf21 levels (Figure 2(f)) and *Fgf21* mRNA (Figure 3(d)) in male mice.

Seladelpar treatment also increased liver alkaline phosphatase mRNA (Figures S2C and S2D) expression in both female and male mice. No changes in the mRNA expression levels of *Cyp27a1* (Figures 3(e) and 3(f)), the rate-limiting enzyme for the alternative bile acid synthetic pathway, albumin (Figures S2A and S2B), and 5⁣′-nucleotidase mRNA (Figures S2E and S2F) were observed in control or seladelpar-treated mice of either sex. There were small but apparent differences in *Cyp8b1* following multiple treatments (Figures 3(g) and 3(h)), which may reflect changes in the bile acid pool.

### 3.4. Global Genome-Wide Transcriptomic Changes in Liver

**Figure 4:**
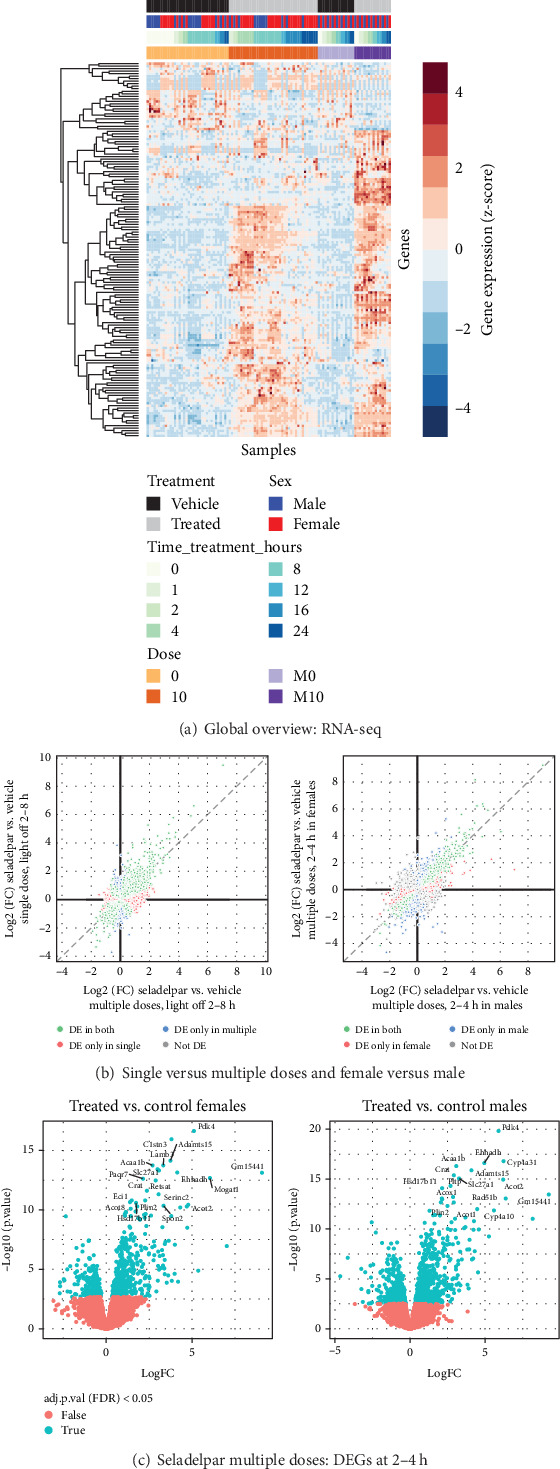
Global RNA-seq changes in the liver of mice following seladelpar treatment. (a) The heat map shows globally upregulated/downregulated genes in female and male mice following single- and multiple-dose seladelpar treatment (lights-on/off). (b) Scatterplots showing the correlation between DEGs from single and multiple doses of seladelpar treatment (left, light-off 2–8 h) and between females and males (right, 2–4 h). (c) A volcano plot comparing DEGs (2–4 h) in control and multiple-dose seladelpar-treated female or male mice. DE, differentially expressed; DEGs, differentially expressed genes; h, hours.

Genome-wide RNA-seq was performed to examine the effects of seladelpar treatment on transcriptomics in the liver. RNA libraries were pooled for efficiency, and this did not affect data interpretation. A single pooled sample was analyzed for all time points except on Day 1, where 0- and 8-h samples from individual animals were analyzed separately (Figure S1). Principal component (PC) analysis on scaled data revealed that the samples clustered based on sex (male/female), dose (single/multiple doses), collection time, and treatment (control/seladelpar), and no outliers were detected. Principal Component 1 (PC1), which explained approximately 27% of the variance in gene expression, was significantly correlated to sex and dose (Kruskal–Wallis test, *p* value < 0.01) (Figures S3A, S3B, and S3E). Principal Component 2 (PC2), which explained approximately 12% of the variance, was correlated to the time of sample collection and treatment (Figures S3C–S3E). Different time points were used as replicates for differential expression analysis, and sex and/or time were used as covariates based on the contrasts assessed. To calculate differential gene expression upon seladelpar treatment on Day 1 and Day 8 (single and multiple doses) during the light and dark cycles, three timeframes were chosen: 0–1 h (lights-on 1), 2–8 h (lights-off), and 12–24 h (lights-on 2), and time and sex were used as covariates. A heat map showing globally upregulated and downregulated genes in the liver of control and seladelpar-treated mice is provided in Figure 4(a). A total of 149 differentially expressed genes (DEGs) were identified in the liver following single or multiple doses of seladelpar treatment or both (absolute value of log2FC (abs[log2FC]) > 2 and a false discovery rate < 0.01). Of them, 59 DEGs were common for single or multiple doses of seladelpar, 13 DEGs were common for the single dose alone, and 77 DEGs were common for multiple doses alone. To evaluate sex-specific expression changes upon treatment, four timeframes were chosen: 0–1 h, 2–4 h, 8–12 h, and 16–24 h for each dose type and sex, and time was used as a covariate.

A strong correlation was observed between the DEGs following single- and multiple-dose seladelpar treatment (Figure 4(b), left, light-off 2–8 h). However, certain DEGs showed no correlation between single and multiple doses. A similar trend was observed when female mice were compared with male mice, with a high similarity among the top DEGs (Figure 4(b), right, 2–4 h). After single-dose seladelpar administration, DEGs increased from 2–4 h to 8–12 h and decreased from 16 to 24 h. Multiple doses induced strong DEGs in the liver at all the time points analyzed (0–1, 2–4, 8–12, and 16–24 h), indicated by a robust correlation between time points (data not shown). A representative image showing the DEGs in female and male mice at 2–4 h following multiple-dose seladelpar treatment is given in Figure 4(c).

### 3.5. Seladelpar Treatment Induced the Expression of PPAR-Modulated Genes

**Figure 5:**
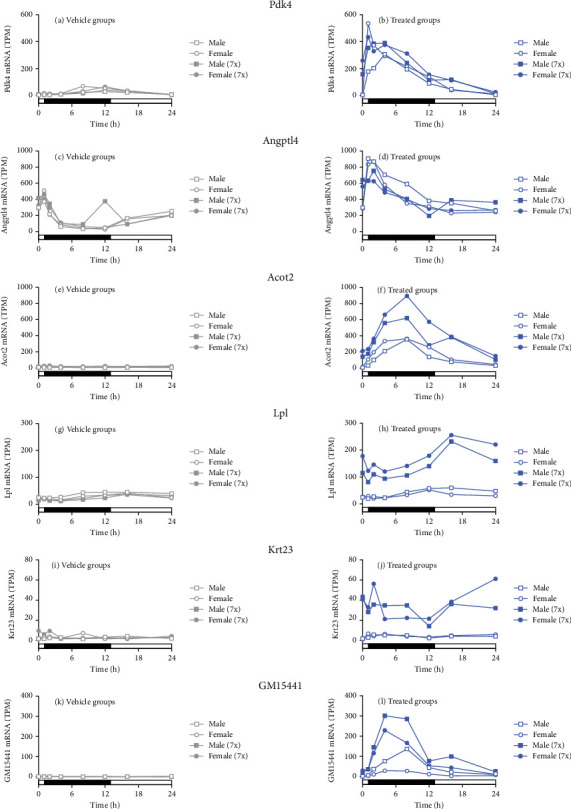
Seladelpar treatment–induced expression of PPAR-modulated genes. *Pdk4*, *Angptl4*, *Acot2*, *Lpl*, *Krt23*, and *GM15441* expression in (a, c, e, g, i, k) control (gray circles for females and gray squares for males) and (b, d, f, h, j, l) single- or multiple-dose seladelpar-treated female or male mice (blue circles for females and blue squares for males). Open symbols denote a single dose of seladelpar treatment or vehicle administration on Day 1 (1x). Multiple doses of seladelpar or vehicle administration from Day 1 to Day 7 (7x) are denoted using closed symbols. The *y*-axis indicates TPM. TPM, transcripts per million.

RNA-seq showed that prototypical PPAR*δ*-responsive genes pyruvate dehydrogenase kinase 4 (*Pdk4*) and acyl-CoA thioesterase 2 (*Acot2*) were induced immediately following single and multiple doses of seladelpar treatment (Figures 5(b) and 5(f)). Similarly, Angiopoietin-Like 4 (*Angptl4*) expression was upregulated by single and multiple doses of seladelpar (Figure 5(d)). Despite the global correlation between single and multiple doses, some genes, such as lipoprotein lipase (*Lpl*) and keratin 23 (*Krt23*), were upregulated only upon multiple-dose seladelpar treatment in both sexes (Figures 5(h) and 5(j)). Other genes, such as long noncoding RNA (*Gm15441*), demonstrated additive effects with multiple doses of seladelpar (Figure 5(l)).

### 3.6. Seladelpar Did Not Alter Clock Genes, Nuclear Receptors, and Sexually Dimorphic Genes

**Figure 6:**
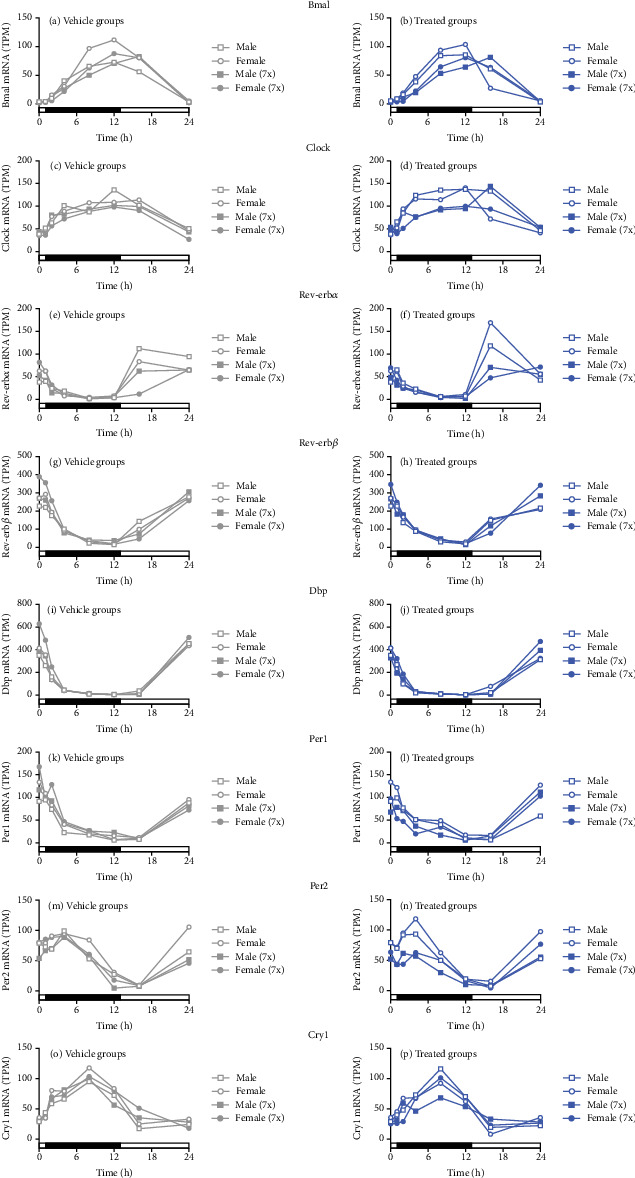
Seladelpar treatment does not affect clock genes. *Bmal*, *Clock*, *Rev-erbα*, *Rev-erbβ*, *Dbp*, *Per1* and *2*, and *Cry1* expression in (a, c, e, g, i, k, m, o) control (gray circles for females and gray squares for males) and (b, d, f, h, j, l, n, p) single- or multiple-dose seladelpar-treated female or male mice (blue circles for females and blue squares for males). Open symbols denote a single dose of seladelpar treatment or vehicle administration on Day 1 (1x). Multiple doses of seladelpar or vehicle administration from Day 1 to Day 7 (7x) are denoted using closed symbols. The *y*-axis indicates TPM. h, hours; TPM, transcripts per million.

Seladelpar treatment did not affect nuclear receptors or clock genes. Examples of genes that were unchanged included *Bmal*, clock circadian regulator (*Clock*), nuclear receptor subfamily 1 group D member 1 and 2 (*Rev-erbα* and *Rev-erbβ*), D-box binding PAR bZIP transcription factor (*Dbp*), *Per1* and *Per2*, and *Cry1* (Figure 6).

Other genes such as peroxisome proliferator–activated receptor alpha (*Pparα*), *Pparδ*, and peroxisome proliferator–activated receptor gamma (*Pparγ*), thyroid hormone receptor alpha and beta (*Thrα* and *Thr**β***), farnesoid X receptor 1 and 2 (*Fxr1* and *Fxr2*), and retinoid X receptor alpha, beta, and gamma (*Rxrα*, *Rxrβ*, and *Rxrγ*) did not change following seladelpar treatment (Figure S4). Also, sex-specific genes such as X-inactive specific transcript (*Xist*) and DEAD-box helicase 3-Y linked (*Ddx3y*) were not altered (Figure S5). Thioredoxin interacting protein (*Txnip*) and Sirtuin 1 (*Sirt1*) gene expression are shown in Figure S6.

## 4. Discussion

This study has demonstrated that seladelpar treatment reduces bile acid synthesis by upregulating Fgf21 and modulating other PPAR-responsive genes. While PPAR*δ* and PPAR*α* may have overlapping targets for a number of genes, it is unlikely that PPAR*δ* and PPAR*α* target the same set of genes in the liver, as hepatic gene expression has been shown to be very different [24, 29] (Figure S4). In light of this, the focus of this study is not the elucidation of the PPAR isoform-specific effects but rather on the investigation of seladelpar and its PPAR*δ* effects over time. A previous study in mice has shown that seladelpar treatment induces Fgf21, and this is a critical component in the decrease in *Cyp7a1* expression at a given specific time in the diurnal cycle [24]. Our current study supports and extends this conclusion by providing a much more complete record of this key regulatory pathway by examining changes over the 24-h cycle in temporal coordination with variations in the global transcriptome. Mice were dosed with seladelpar before feeding to match bile acid synthesis to that in humans.

C4 is a freely diffusible metabolite from the classic bile acid biosynthetic pathway [30, 31]. In this study, C4 levels exhibited a diurnal expression pattern, in accordance with previous observations [3, 10]. Additionally, vehicle-treated mice showed sex-dependent C4 levels, with increased C4 levels in females compared to males. Sexual dimorphism in the enzymes regulating bile synthesis has been previously reported [32]. Also, both outbred and inbred strains of female mice were reported to have an inherently larger bile acid pool compared to male mice [33]. Even though a *Cyp7a1* knockout remarkably decreases the bile acid pool in mice irrespective of sex, female mice maintain a larger bile pool than their male counterparts [34]. This inherent sex-specific difference might have contributed to the distinct response of female mice to single and multiple doses of seladelpar in this study. While the sex-specific C4 levels observed in this study could serve as baseline data for other studies, it also suggests the importance of considering sex-based differences in bile acid metabolism studies.

In this study, single and multiple doses of seladelpar substantially increased plasma Fgf21 levels, a negative regulator of bile acid synthesis [35]. Fgf21 had been reported to be upregulated in humans following the administration of PPAR*α* and PPAR*δ* agonists [36] as well as after seladelpar administration in mice [24]. In a recent study, seladelpar treatment decreased *Cyp7a1* with a simultaneous increase in *Fgf21* in mice, both of which were abolished following *Pparδ* knockdown, suggesting that Fgf21 is induced through Ppar*δ* activation [24]. Additionally, treatment of primary mouse hepatocytes with recombinant Fgf21 protein repressed *Cyp7a1* and activated the c-Jun N-terminal kinase (JNK) signaling pathway. Blockade of the JNK pathway eliminated the repressive effect of seladelpar on *Cyp7a1*, suggesting that seladelpar exerts its effects via Fgf21 upregulation and activation of the JNK signaling pathway [24]. Though JNK pathway proteins were not measured in this study, the temporal patterns of Fgf21 upregulation and *Cyp7a1* and C4 reductions are consistent with Fgf21-mediated JNK signaling.

The high-throughput RNA-seq approach was used to characterize the transcriptomic landscape of the liver in control and seladelpar-treated animals. Despite some differences, the transcriptomic analysis revealed a strong overall correlation between DEGs following single- and multiple-dose seladelpar treatment, suggesting that there are no cumulative effects from repeated exposure to seladelpar. PPAR-responsive genes, *Pdk4*, *Acot2*, and *Angptl4*, were upregulated in this study after seladelpar treatment. Previous research has suggested the role of the FXR (farnesoid X receptor) in hepatic *Pdk4* upregulation [37–39]. However, seladelpar has been shown to function independently of the FXR pathway [24]. Besides, PPARs can directly bind to specific DNA sequences known as PPAR response elements in the promoter regions of the target genes and modulate their activity [40, 41]. Furthermore, PPAR-responsive genes have been reported to be a direct target of PPAR ligands. PPAR*β*/*δ* has been shown to directly bind to *PDK2*, *PDK3*, and *PDK4* genes [42]; PPAR*γ* to the promoter region of *ANGPTL4* [43]; and PPAR*α* to the *Acot1* gene promoter [44]. Therefore, it is likely that seladelpar directly targeted PPAR-responsive genes and modulated their expression.

Another interesting observation was the modulation of *Gm15441* after seladelpar treatment, a natural antisense long noncoding RNA transcribed from the reverse strand of *Txnip*, which is a crucial metabolic regulator in the liver. Multiple doses of seladelpar had an additive effect on *Gm15441*. A previous study has reported that *Txnip* is a PPAR*α* target gene, and its expression is inversely regulated with that of *Gm15441* after PPAR*α* activation [45]. On the other hand, in vitro and in vivo metabolic signals have been shown to modulate *Gm15441* in the liver in a similar pattern to its sense gene *Txnip* [46], which is in line with this study where a similar expression pattern for both *Gm15441* and *Txnip* was observed after seladelpar treatment. The temporal expression of the core clock genes and nuclear receptors was not altered following seladelpar treatment in this study, implying that seladelpar did not affect any of these regulatory mechanisms. Assessment of the clock (*Clock* and *Bmal*) and sex-specific genes (*Xist* and *Ddx3y*) is important as they can serve as internal controls for in vivo studies to assess sampling time and correct study execution.

## 5. Conclusions

In summary, data from this seladelpar study demonstrates predictable and reproducible changes in bile acid synthesis and Fgf21 signaling congruent with the data from the ongoing and completed clinical trials. In a Phase 3 study in patients with primary biliary cholangitis [47], seladelpar treatment has been shown to increase serum Fgf21 while decreasing serum C4 and total bile acid levels [47]. The results from the current mouse study provide novel and translatable mechanistic insights into human data [47]. A single dose of a PPAR*δ* agonist reveals important molecular consequences of PPAR*δ*-mediated changes in gene expression. Sex-based differences in bile acid synthesis in mice warrant further investigation in humans to confirm and extend the limited reports of cycling and diurnal variation in males [3]. Extending these findings to understanding the molecular mechanisms in model systems and applying them to human biology, in both health and disease, will be important to understanding the role of bile and biliary recycling and discovering and developing better therapeutics.

## Data Availability

Data will be made available upon reasonable request.

## Nomenclature

Acc: acetyl-CoA carboxylase
Acot: acyl-CoA thioesterase
Angptl: angiopoietin-like
Bmal: basic helix-loop-helix ARNT-like
Clock: circadian locomotor output cycles kaput
Cry: cryptochrome circadian regulator
Cyp: cytochrome P450
Dbp: D-box binding PAR bZIP transcription factor
Ddx3y: DEAD-box helicase 3-Y linked
DEG: differentially expressed genes
EDTA: ethylenediaminetetraacetic acid
ELISA: enzyme-linked immunosorbent assay
Fasn: fatty acid synthase
FC: fold change
Fgf: fibroblast growth factor
Fxr: farnesoid X receptor
JNK: c-Jun N-terminal kinase
Krt: keratin
Limma: linear models for microarray data
Lpl: lipoprotein lipase
PC: principal component
Per: period circadian regulator
Pdk: pyruvate dehydrogenase kinase
PPAR: peroxisome proliferator–activated receptors
Rev-erb: nuclear receptor subfamily 1 group D member
Rxr: retinoid X receptor
Scd: stearoyl-CoA
SEM: standard error mean
Sirt: sirtuin
Thr: thyroid hormone receptor
TPM: transcripts per million
Txnip: thioredoxin interacting protein
Xist: X-inactive specific transcript
