# Supplementary material for: Single and Multiple Doses of Seladelpar Decrease Diurnal Markers of Bile Acid Synthesis in Mice
Source: PPAR Res. 2025 Mar 11;2025:5423221. doi: 10.1155/ppar/5423221 (PMC11991775; doi:10.1155/ppar/5423221)
Supplement: Supporting Information — Additional supporting information can be found online in the Supporting Information section. Figures S1–S6. [file 5423221.f1.docx]

**Title:** Single and multiple doses of seladelpar decrease diurnal markers of bile acid synthesis in mice

**Running title**: Seladelpar decreases bile acid synthesis

**Authors:** Edward E. Cable,^1^ Jeffrey W. Stebbins,^1^ Jeff D. Johnson,^1^ Yun-Jung Choi,^1^ Jiangao Song,^1^ Sole Gatto,^2^ Matthew Onorato,^2^ Charles A. McWherter^1^

**Affiliations:**

^1^CymaBay Therapeutics, Inc., [7601 Dumbarton Cir, Fremont, CA 94555, United States](https://www.google.com/maps/place/data=!4m2!3m1!1s0x808fbe603abca46d:0xa8f0280f0a2c95c7?sa=X&ved=1t:8290&ictx=111)

^2^Monoceros Biosystems LLC, 12636 High Bluff Drive, Suite 400, San Diego, CA 92130, United States

**Corresponding author:**

Edward E. Cable

Senior Director of Research

CymaBay Therapeutics, Inc.

Fremont, CA 94555, United States

Tel: +1-619-254-1364

ORCID ID: [https://orcid.org/0000-0002-9268-968X](https://nam11.safelinks.protection.outlook.com/?url=https%3A%2F%2Forcid.org%2F0000-0002-9268-968X&data=05%7C02%7Cshereen.dcruz%40certara.com%7C247e64c2e5694c86531508dc6473ae3b%7C7287abd30220456e98514352bae208c9%7C1%7C0%7C638495695230301923%7CUnknown%7CTWFpbGZsb3d8eyJWIjoiMC4wLjAwMDAiLCJQIjoiV2luMzIiLCJBTiI6Ik1haWwiLCJXVCI6Mn0%3D%7C0%7C%7C%7C&sdata=QK8GaqSvvlDSH7aEtO0hUdzPdryaw4hNtlGWFjshZCc%3D&reserved=0)

Due to the recent acquisition of CymaBay Therapeutics Inc., the corresponding author, Edward E. Cable, can be contacted at:

333 Lakeside Drive

Foster City, CA 94404

cable.ed@gmail.com

**SUPPLEMENTAL FIGURES**


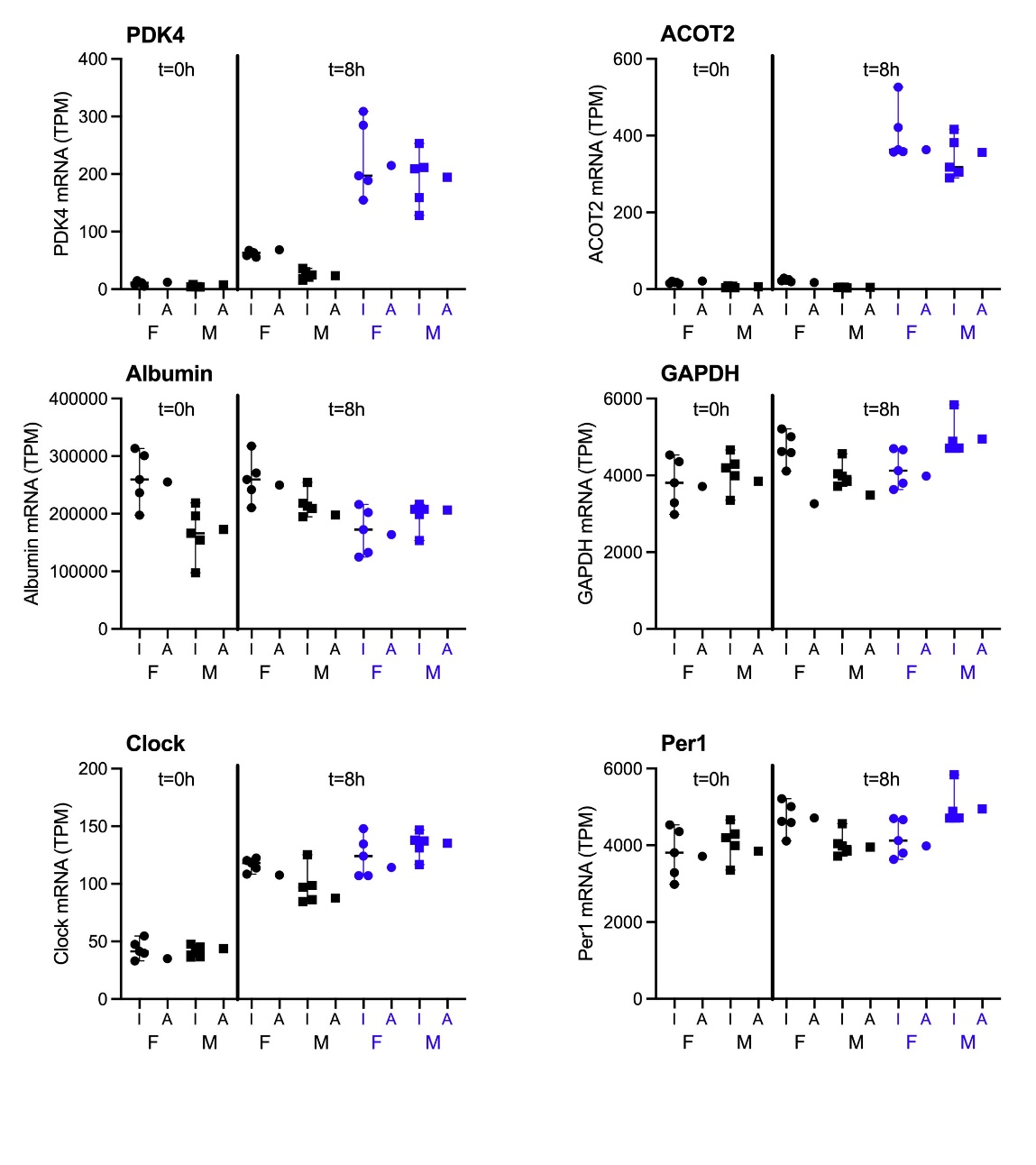


**Supplemental Figure S1.** The data show all 5 individual points with medians±95% CI (I) versus the bioaveraged samples. The bioaveraged (A) samples used equal amounts of input mRNA from each mouse to generate the library for RNA-seq. The data shown are t=0h, vehicle only, and t=8h for both vehicle (black) and treated (blue) mice. F and M are indicated by circles and squares, respectively. The selected graphs include 2 seladelpar‑inducible genes, 2 housekeeping genes, and 2 clock genes. The bioaveraging produced data consistent with n=5 for each group.

CI, confidence interval; F, female; h, hours; M, male; t, time; TPM, transcripts per million

**
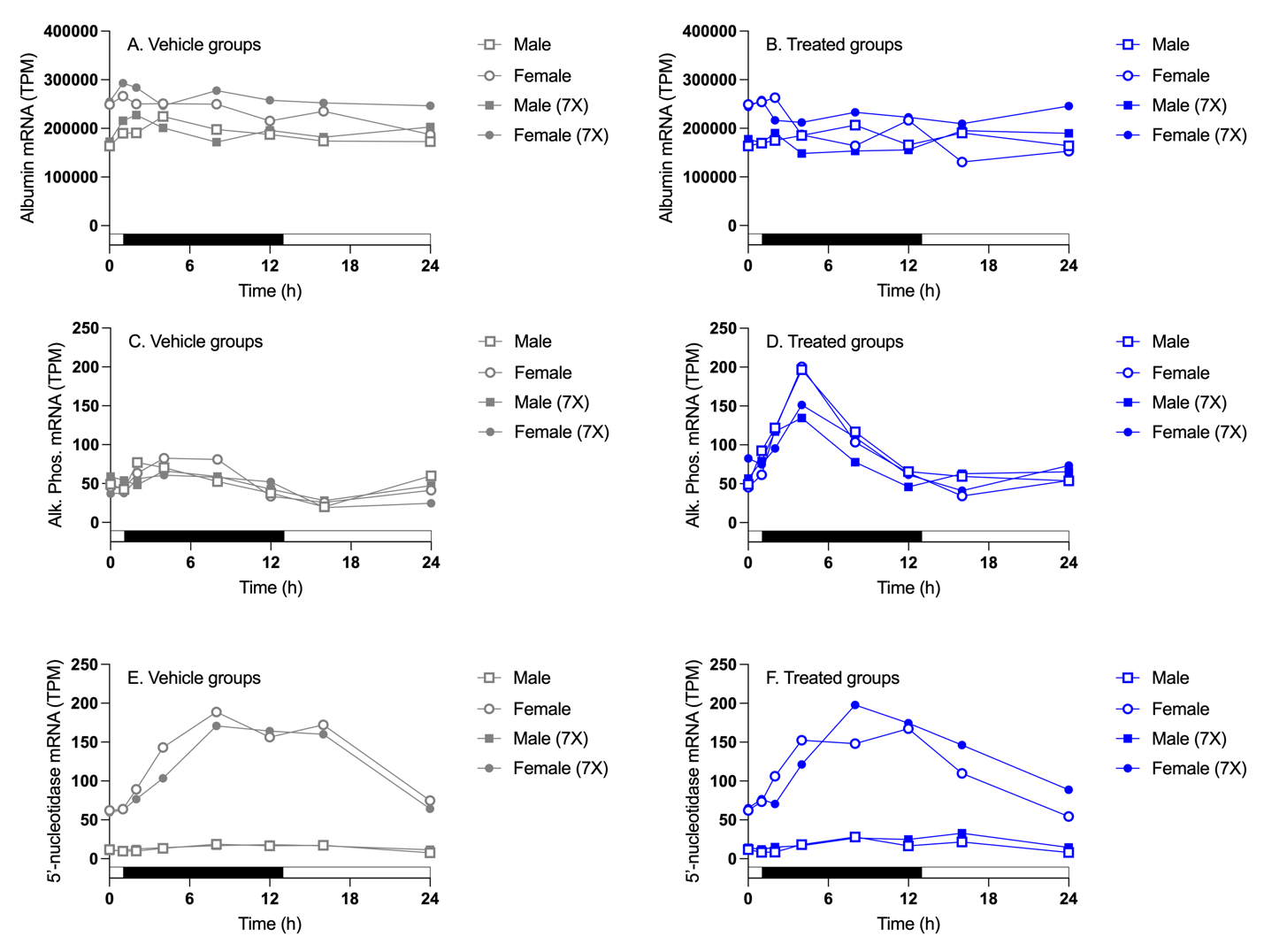
**

**Supplemental Figure S2.** Albumin, alkaline phosphatase, and 5’-nucleotidase expression in control (A, C, E; gray circles for females and gray squares for males; open symbols for Day 1 [1x] and closed symbols for Day 1 to Day 7 [7x]) and single‑ or multiple‑dose seladelpar-treated female or male mice (B, D, F; blue circles for females and blue squares for males; open symbols for single dose on Day 1 [1x] and closed symbols for multiple doses from Day 1 to Day 7 [7x]). Gamma glutamyl-transferase expression was below the limit of detection for about half of the samples (data not shown). The y-axis indicates TPM.

h, hours; TPM, transcripts per million


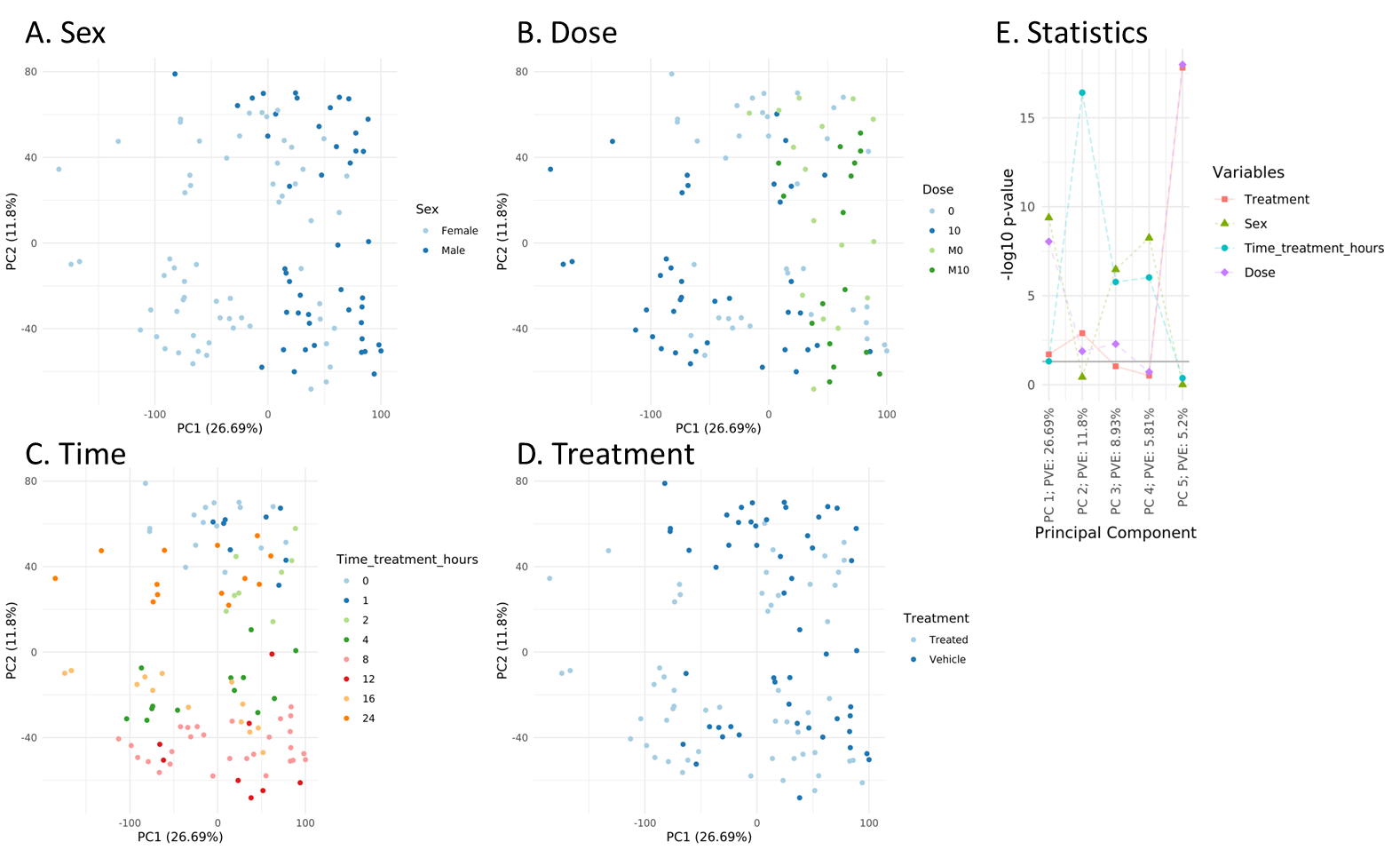


**Supplemental Figure S3.** PC analysis showing correlation to sex (A), dose (B), time of sample collection (C), and treatment (D). Kruskal-Wallis log10(p-value) is plotted to evaluate correlation between each variable versus all principal components (E). P‑value=0.05 is log10(p-value)=1.3.

PC, principal component


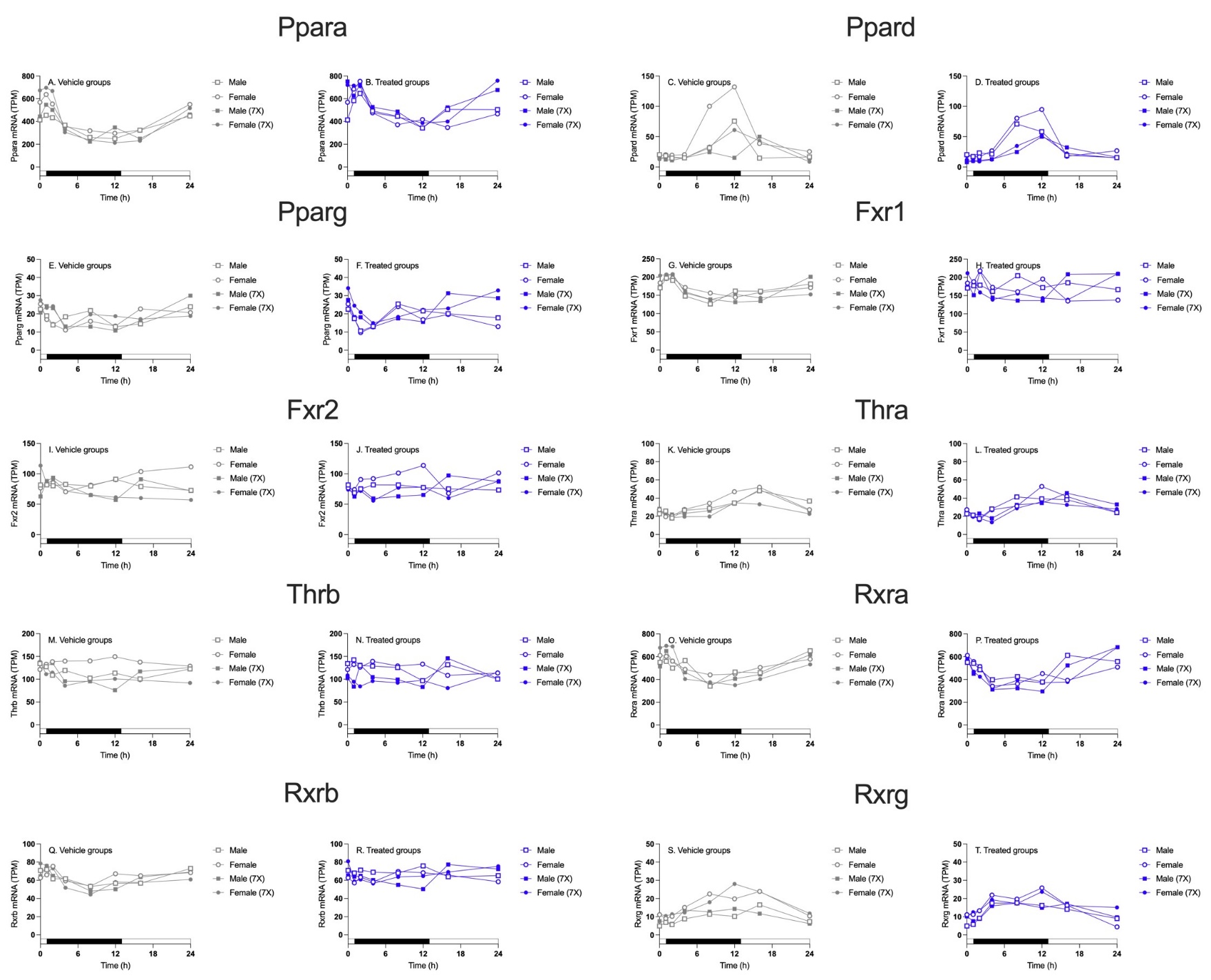


**Supplemental Figure S4.** Nuclear receptor gene expression in control (gray circles for females and gray squares for males; open symbols for Day 1 [1x] and closed symbols Day 1 to Day 7 [7x]) and single‑ or multiple‑dose seladelpar-treated female or male mice (blue circles for females and blue squares for males; open symbols for single dose on Day 1 [1x] and closed symbols for multiple doses from Day 1 to Day 7 [7x]). The y-axis indicates TPM.

h, hours; TPM, transcripts per million

**
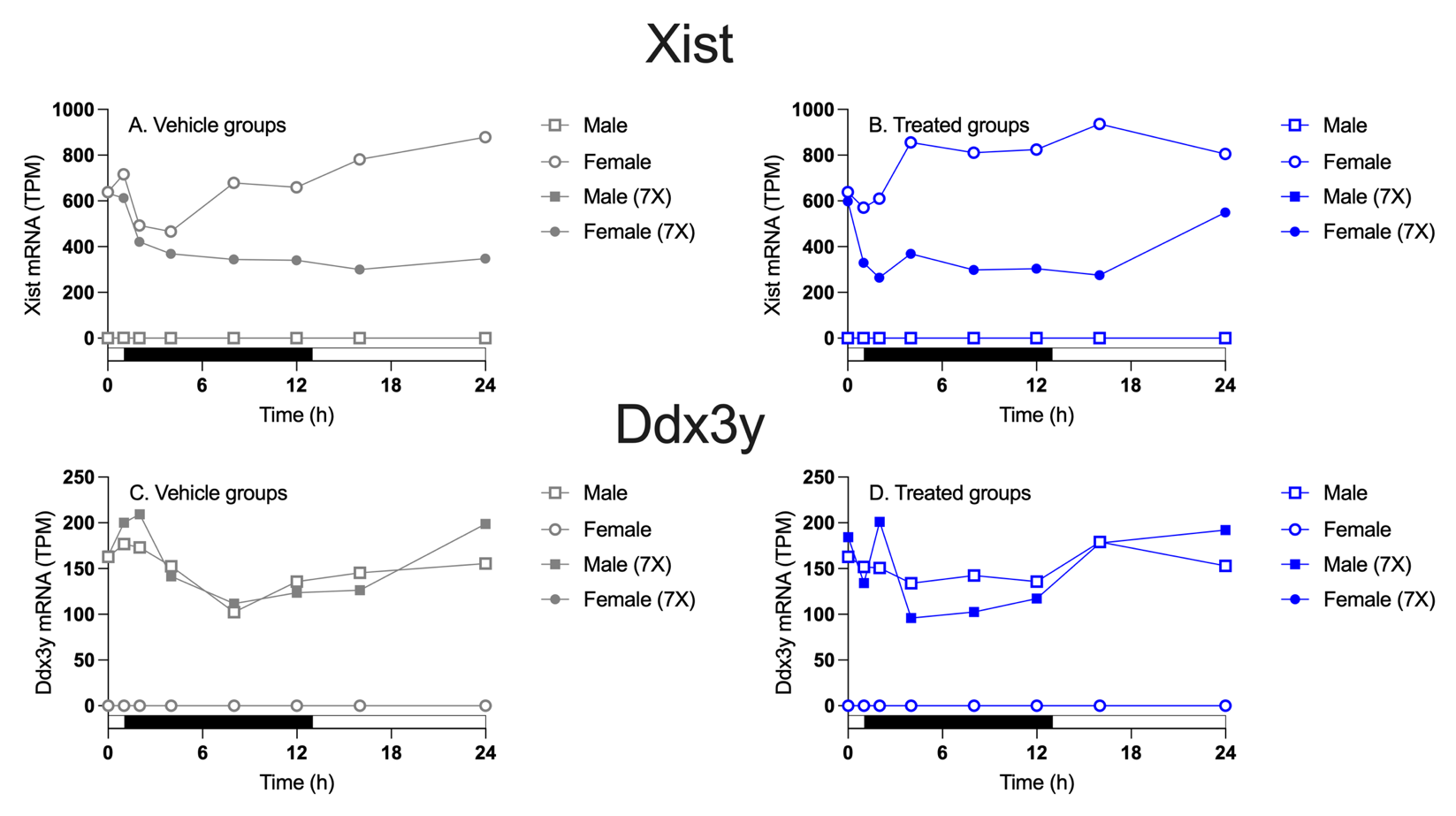
**

**Supplemental Figure S5.** Gene expression of sex-specific genes, *Xist* and *Ddx3y*, in control (A, C; gray circles for females and gray squares for males; open symbols for Day 1 [1x] and closed symbols Day 1 to Day 7 [7x]) and single‑ or multiple‑dose seladelpar‑treated female or male mice (B, D; blue circles for females and blue squares for males; open symbols for single dose on Day 1 [1x] and closed symbols for multiple doses from Day 1 to Day 7 [7x]). The y-axis indicates TPM.

h, hours; TPM, transcripts per million

**
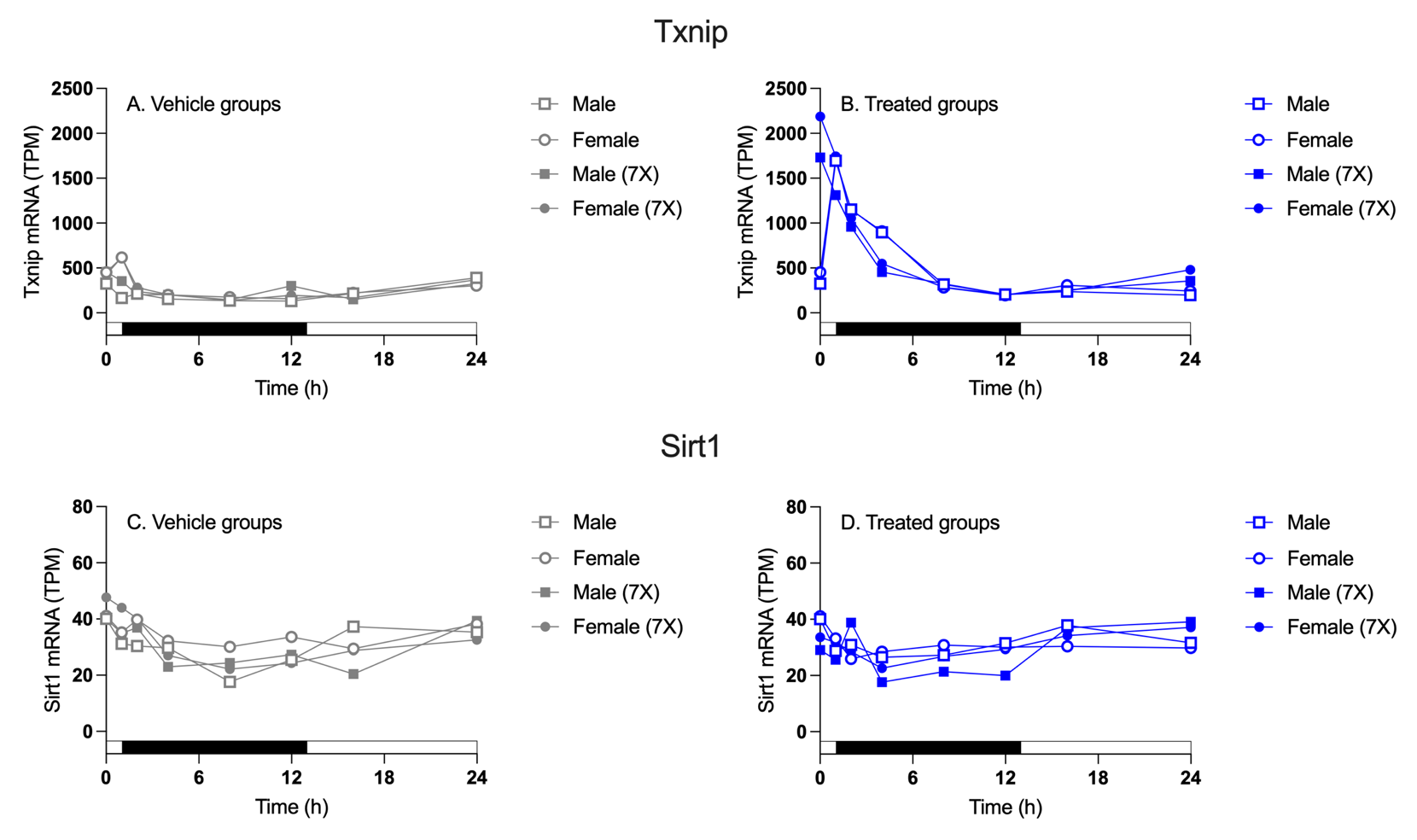
**

**Supplemental Figure S6.** *Txnip* and *Sirt1* gene expression in control (A, C; gray circles for females and gray squares for males; open symbols for Day 1 [1x] and closed symbols Day 1 to Day 7 [7x]) and single‑ or multiple‑dose seladelpar-treated female or male mice (B, D; blue circles for females and blue squares for males; open symbols for single dose on Day 1 [1x] and closed symbols for multiple doses from Day 1 to Day 7 [7x]). The y-axis indicates TPM.

h, hours; TPM, transcripts per million
